# Effects of acetoacetyl-CoA synthase expression on production of farnesene in *Saccharomyces cerevisiae*

**DOI:** 10.1007/s10295-017-1911-6

**Published:** 2017-02-09

**Authors:** Stefan Tippmann, Raphael Ferreira, Verena Siewers, Jens Nielsen, Yun Chen

**Affiliations:** 1 Department of Biology and Biological Engineering, Chalmers University of Technology, Kemivägen 10, SE412 96 Gothenburg, Sweden; 2 Novo Nordisk Foundation Center for Biosustainability, Chalmers University of Technology, SE412 96 Gothenburg, Sweden; 3 Novo Nordisk Foundation Center for Biosustainability, Technical University of Denmark, DK2800 Kongens Lyngby, Denmark

**Keywords:** Isoprenoids, Mevalonate pathway, Biofuels, Yeast, Metabolic engineering

## Abstract

Efficient production of sesquiterpenes in *Saccharomyces cerevisiae* requires a high flux through the mevalonate pathway. To achieve this, the supply of acetyl-CoA plays a crucial role, partially because nine moles of acetyl-CoA are necessary to produce one mole of farnesyl diphosphate, but also to overcome the thermodynamic constraint imposed on the first reaction, in which acetoacetyl-CoA is produced from two moles of acetyl-CoA by acetoacetyl-CoA thiolase. Recently, a novel acetoacetyl-CoA synthase (*nphT7*) has been identified from *Streptomyces* sp. strain CL190, which catalyzes the irreversible condensation of malonyl-CoA and acetyl-CoA to acetoacetyl-CoA and, therefore, represents a potential target to increase the flux through the mevalonate pathway. This study investigates the effect of acetoacetyl-CoA synthase on growth as well as the production of farnesene and compares different homologs regarding their efficiency. While plasmid-based expression of *nphT7* did not improve final farnesene titers, the construction of an alternative pathway, which exclusively relies on the malonyl-CoA bypass, was detrimental for growth and farnesene production. The presented results indicate that the overall functionality of the bypass was limited by the efficiency of acetoacetyl-CoA synthase (*nphT7*). Besides modulation of the expression level, which could be used as a means to partially restore the phenotype, *nphT7* from *Streptomyces glaucescens* showed clearly higher efficiency compared to *Streptomyces* sp. strain CL190.

**Electronic supplementary material:**

The online version of this article (doi:10.1007/s10295-017-1911-6) contains supplementary material, which is available to authorized users.

## Introduction

**Fig. 1:**
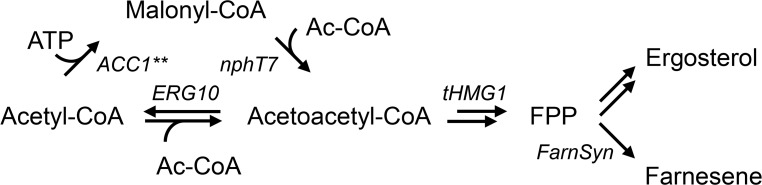
Simplified illustration of the mevalonate pathway in *S. cerevisiae*, which is utilized for sterol synthesis. Besides, farnesyl diphosphate (FPP) can be converted to farnesene. In the first reaction of the pathway, acetoacetyl-CoA thiolase (*ERG10*) produces acetoacetyl-CoA from two molecules of acetyl-CoA. Alternatively, acetoacetyl-CoA can be produced from malonyl-CoA using acetoacetyl-CoA synthase (*nphT7*) identified from *Streptomyces* sp. strain CL190, which was investigated in this study

The mevalonate pathway is of crucial importance for *Saccharomyces cerevisiae* as it is used for synthesis of sterols, ubiquinone and dolichols. Besides, the pathway has been exploited for the production of sesquiterpenes, which have different applications within the chemical industry and can be provided from microbial fermentation using renewable carbon sources [18]. In the first reaction of the pathway, acetoacetyl-CoA thiolase (encoded by *ERG10*) catalyzes the reversible non-decarboxylative Claisen condensation of two acetyl-CoA molecules to produce acetoacetyl-CoA (Fig. 1). Activity of this enzyme was shown to be regulated by intracellular sterol levels [7, 9], whereas loss of *ERG10* was reported to result in mevalonate auxotrophy [10]. However, the reaction is thermodynamically unfavorable (Δ*G* ^0^′ ≈ 7 kcal/mol [14, 34]), imposing constraints on the concentrations of acetyl-CoA to enable acetoacetyl-CoA synthesis. Recently, *nphT7* present in the mevalonate pathway gene cluster in *Streptomyces* sp. strain CL190 was identified to encode a novel acetoacetyl-CoA synthase [21], which catalyzes the energetically more favorable (estimated Δ*G* ^0^′ = −0.9 kcal/mol [34]) decarboxylative condensation of malonyl-CoA and acetyl-CoA to acetoacetyl-CoA. The enzyme was reported to produce acetoacetyl-CoA also from malonyl-CoA as sole substrate, but most importantly, it did not display thiolysis activity in vitro [21]. For this reason, it was suggested to be ideal for the production of butanol, polyhydroxyalkanoates and isoprenoids. In *S. cerevisiae*, the enzyme successfully supported butanol production and also displayed similar enzymatic activity in vitro in comparison to the endogenous acetoacetyl-CoA thiolase [29]. In contrast, Menon et al. reported 6.2-fold higher titers of butanol produced from *Escherichia coli* using the endogenous acetoacetyl-CoA thiolase (*atoB*) over *nphT7* for supply of acetoacetyl-CoA [19]. Furthermore, acetoacetyl-CoA synthase has also been used for the production of butanol in *Synechococcus elongatus* [14], for the production of propane in *E. coli* [19] and for poly-3-hydroxybutyrate production in mesophyllic cells of sugarcane [17]. The objective of this study was to investigate the effect of the acetoacetyl-CoA synthase on the production of sesquiterpenes in *S. cerevisiae* using farnesene as an example. Farnesene is directly produced from farnesyl diphosphate (FPP) using farnesene synthase and its production requires 9 mol∙mol^−1^ of acetyl-CoA. Efficient conversion of acetyl-CoA to acetoacetyl-CoA using *nphT7* for increased flux through the pathway represents a potential target to improve the production of these compounds. Since the enzyme requires malonyl-CoA as extender substrate, overexpression of the modified acetyl-CoA carboxylase (ACC1**), which displayed enhanced activity, was used additionally to promote the influx from malonyl-CoA into the pathway [30]. As increased levels of acetoacetyl-CoA could potentially stimulate acetoacetyl-CoA thiolysis and result in a futile cycle, the endogenous *ERG10* was replaced by *nphT7* as an alternative approach to produce acetoacetyl-CoA exclusively from malonyl-CoA and to compare both pathways regarding growth and product formation. Since carboxylation of acetyl-CoA to malonyl-CoA requires additional ATP, which could reduce the product yield, this route becomes less efficient with respect to free energy conservation (Fig. 1). However, energy released from ATP hydrolysis (Δ*G* ^0^′ < −7 kcal/mol) could be utilized successfully to drive biochemical reactions for butanol production, by compensating for the required energy for acetoacetyl-CoA synthesis via condensation of two molecules of acetyl-CoA [14].

## Materials and methods

### Plasmid and strain construction

The *nphT7* gene from *Streptomyces* sp. strain CL190 (*nphT7*_*SCL*_) as well as the homologous genes from *Streptomyces glaucescens*, *Streptomyces afghaniensis*, *Streptomyces lactacystinaeus* and *Nocardia brasiliensis* were codon optimized and synthesized by GenScript (Piscataway, NJ, USA). *nphT7*_*SCL*_ was amplified by PCR and cloned into pSP-GM1 using restriction enzymes *Not*I and *Pac*I, resulting in plasmid pIST07. For construction of plasmids pIST12-16, the truncated *HXT7* promoter P_*tHXT7*_ and *HIS5* terminator T_*HIS5*_ were amplified from *S. cerevisiae* CEN.PK113-5D and fused to each of the *nphT7* genes by PCR. The constructed cassettes were then amplified by PCR to generate the complementary overhangs for insertion into plasmid pIST05 using CPEC [25]. An overview of all plasmids used in this study is provided by Table 1. Primers used for plasmid construction are listed in Table S1. Chromosomal integration of ACC1** [30] into site X-2 was achieved using plasmid pMG96 (provided by Dr. Michael Gossing, Chalmers University of Technology, Sweden), a derivative of vector pCfB353 [11]. Substitutions of *ERG10* by *nphT7* from *Streptomyces* sp. strain CL190 were performed in strains RF14 and IMX581 using CRISPR/Cas9 [15]. IMX581 and the CRISPR plasmid backbone (pMEL10) were obtained from EUROSCARF (Frankfurt, Germany). The guide RNA targeting the *ERG10* locus was designed using the Yeastriction webtool (http://yeastriction.tnw.tudelft.nl/). The *nphT7*_*SCL*_ gene was amplified from pIST07 by PCR and used as repair fragment. Simultaneous transformation with pMEL10, the *ERG10* specific guide RNA and the *nphT7*_*SCL*_ repair fragment resulted in the in vivo assembly of the plasmid, mediating a double-strand cut in the *ERG10* gene by the Cas9 nuclease. The double-strand break allowed integration of the *nphT7*_*SCL*_ cassette by homologous recombination, ultimately resulting in strain SCIST17, 19 (*nphT7* expression using endogenous promoter and terminator) and SCIST38 (*nphT7*_*SCL*_ expression using P_*TEF1*_/T_*ADH1*_). Replacing the endogenous *FAS1* promoter P_*FAS1*_ by P_*HXT1*_ for construction of strain SCIST41 was performed as described previously [5]. Table 2 provides an overview of all strains used in this study, whereas primers used for construction of these strains are listed in Table S2.

**Table 1:** List of plasmids used in this study

Plasmid	Description	References
pSP-GM1	P_*TEF1*_-P_*PGK1*_ bidirectional promoter (2 μm, *URA3*)	[3]
pMEL10	gRNA-*CAN1.*Y (2 μm, *URA3*)	[15]
pIST05	P_*PGK1*_-*tHMG1*, P_*TEF1*_-*FarnSyn_Cj*	[32]
pMG96	P_*TEF1*_-ACC1**	This study
pIST07	P_*TEF1*_- *nphT7*_*SCL*_	This study
pIST12	pIST05, P_*tHXT7*_-*nphT7*_*SCL*_	This study
pIST13	pIST05, P_*tHXT7*_-*nphT7*_*Sg*_	This study
pIST14	pIST05, P_*tHXT7*_-*nphT7*_*Sa*_	This study
pIST15	pIST05, P_*tHXT7*_-*nphT7*_*Sl*_	This study
pIST16	pIST05, P_*tHXT7*_-*nphT7*_*Nb*_	This study

**Table 2:** List of strains used in this study

Name	Relevant Genotype	Plasmid	References
CEN.PK113-5D	*MAT*a *MAL2*-*8* ^*c*^ *SUC2 ura3*-*52*	–	P. Kötter, University of Frankfurt, Germany
SCIST05	*MAT*a *MAL2*-*8* ^*c*^ *SUC2 ura3*-*52*	pIST05	[32]
IMX581	*MAT*a *MAL2*-*8* ^*c*^ *SUC2 ura3*-*52 can1*∆::*cas9*-*natNT2*	–	[15]
SCIST15	*MAT*a *MAL2*-*8* ^*c*^ *SUC2 ura3*-*52 can1*∆::*cas9*-*natNT2*	pIST05	This study
RF14	*MAT*a *MAL2*-*8* ^*c*^ *SUC2 ura3*-*52* P_*TEF1*_-ACC1** P_*TEF1*_-*kanMX can1*∆::*cas9*-*natNT2*	–	This study
SCIST16	*MAT*a *MAL2*-*8* ^*c*^ *SUC2 ura3*-*52* P_*TEF1*_-ACC1** P_*TEF1*_-*kanMX can1*∆::*cas9*-*natNT2*	pIST05	This study
SCIST17	*MAT*a *MAL2*-*8* ^*c*^ *SUC2 ura3*-*52 can1*∆::*cas9*-*natNT2 erg10*∆::*nphT7*_*SCL*_	–	This study
SCIST18	*MAT*a *MAL2*-*8* ^*c*^ *SUC2 ura3*-*52 can1*∆::*cas9*-*natNT2 erg10*∆:: *nphT7*_*SCL*_	pIST05	This study
SCIST19	*MAT*a *MAL2*-*8* ^*c*^ *SUC2 ura3*-*52* P_*TEF1*_-ACC1** *can1*∆::*cas9*-*natNT2 erg10*∆:: *nphT7*_*SCL*_	–	This study
SCIST20	*MAT*a *MAL2*-*8* ^*c*^ *SUC2 ura3*-*52* P_*TEF1*_-ACC1** *can1*∆::*cas9*-*natNT2 erg10*∆:: *nphT7*_*SCL*_	pIST05	This study
SCIST21	*MAT*a *MAL2*-*8* ^*c*^ *SUC2 ura3*-*52*	pIST12	This study
SCIST22	*MAT*a *MAL2*-*8* ^*c*^ *SUC2 ura3*-*52*	pIST13	This study
SCIST23	*MAT*a *MAL2*-*8* ^*c*^ *SUC2 ura3*-*52*	pIST14	This study
SCIST24	*MAT*a *MAL2*-*8* ^*c*^ *SUC2 ura3*-*52*	pIST15	This study
SCIST25	*MAT*a *MAL2*-*8* ^*c*^ *SUC2 ura3*-*52*	pIST16	This study
SCIST26	*MAT*a *MAL2*-*8* ^*c*^ *SUC2 ura3*-*52* P_*TEF1*_-ACC1** P_*TEF1*_-*kanMX*	–	This study
SCIST27	*MAT*a *MAL2*-*8* ^*c*^ *SUC2 ura3*-*52* P_*TEF1*_-ACC1** P_*TEF1*_-*kanMX*	pIST05	This study
SCIST28	*MAT*a *MAL2*-*8* ^*c*^ *SUC2 ura3*-*52* P_*TEF1*_-ACC1** P_*TEF1*_-*kanMX*	pIST12	This study
SCIST29	*MAT*a *MAL2*-*8* ^*c*^ *SUC2 ura3*-*52* P_*TEF1*_-ACC1** P_*TEF1*_-*kanMX*	pIST13	This study
SCIST30	*MAT*a *MAL2*-*8* ^*c*^ *SUC2 ura3*-*52* P_*TEF1*_-ACC1** P_*TEF1*_-*kanMX*	pIST14	This study
SCIST31	*MAT*a *MAL2*-*8* ^*c*^ *SUC2 ura3*-*52* P_*TEF1*_-ACC1** P_*TEF1*_-*kanMX*	pIST15	This study
SCIST32	*MAT*a *MAL2*-*8* ^*c*^ *SUC2 ura3*-*52* P_*TEF1*_-ACC1** P_*TEF1*_-*kanMX*	pIST16	This study
SCIST33	*MAT*a *MAL2*-*8* ^*c*^ *SUC2 ura3*-*52* P_*TEF1*_-ACC1** *can1*∆::*cas9*-*natNT2 erg10*∆:: *nphT7*_*SCL*_	pIST12	This study
SCIST34	*MAT*a *MAL2*-*8* ^*c*^ *SUC2 ura3*-*52* P_*TEF1*_-ACC1** *can1*∆::*cas9*-*natNT2 erg10*∆:: *nphT7*_*SCL*_	pIST13	This study
SCIST35	*MAT*a *MAL2*-*8* ^*c*^ *SUC2 ura3*-*52* P_*TEF1*_-ACC1** *can1*∆::*cas9*-*natNT2 erg10*∆:: *nphT7*_*SCL*_	pIST14	This study
SCIST36	*MAT*a *MAL2*-*8* ^*c*^ *SUC2 ura3*-*52* P_*TEF1*_-ACC1** *can1*∆::*cas9*-*natNT2 erg10*∆:: *nphT7*_*SCL*_	pIST15	This study
SCIST37	*MAT*a *MAL2*-*8* ^*c*^ *SUC2 ura3*-*52* P_*TEF1*_-ACC1** *can1*∆::*cas9*-*natNT2 erg10*∆:: *nphT7*_*SCL*_	pIST16	This study
SCIST38	*MAT*a *MAL2*-*8* ^*c*^ *SUC2 ura3*-*52* P_*TEF1*_-ACC1** *can1*∆::*cas9*-*natNT2 erg10*∆::P_*TEF1*_-*nphT7*_*SCL*_	–	This study
SCIST39	*MAT*a *MAL2*-*8* ^*c*^ *SUC2 ura3*-*52* P_*TEF1*_-ACC1** *can1*∆::*cas9*-*natNT2 erg10*∆::P_*TEF1*_-*nphT7*_*SCL*_	pIST05	This study
SCIST40	*MAT*a *MAL2*-*8* ^*c*^ *SUC2 ura3*-*52* P_*TEF1*_-ACC1** *can1*∆::*cas9*-*natNT2 erg10*∆::P_*TEF1*_-*nphT7*_*SCL*_	pIST13	This study
SCIST41	*MAT*a *MAL2*-*8* ^*c*^ *SUC2 ura3*-*52* P_*TEF1*_-ACC1** *can1*∆::*cas9*-*natNT2 erg10*∆::P_*TEF1*_-*nphT7*_*SCL*_ P_*TEF1*_-*kanMX* P_*FAS1*_∆::P_*HXT1*_	–	This study
SCIST43	*MAT*a *MAL2*-*8* ^*c*^ *SUC2 ura3*-*52* P_*TEF1*_-ACC1** *can1*∆::*cas9*-*natNT2 erg10*∆::P_*TEF1*_-*nphT7*_*SCL*_ P_*TEF1*_-*kanMX* P_*FAS1*_∆::P_*HXT1*_	pIST13	This study

### Growth medium

*Saccharomyces cerevisiae* strains with uracil auxotrophy were grown on YPD plates containing 20 g/L glucose, 10 g/L yeast extract, 20 g/L peptone from casein and 20 g/L agar. Plasmid carrying strains were grown on selective growth medium containing 6.9 g/L yeast nitrogen base w/o amino acids (Formedium, Hunstanton, UK), 0.77 g/L complete supplement mixture w/o uracil (Formedium), 20 g/L glucose and 20 g/L agar. Shake flask cultivations were performed in minimal medium containing 30 g/L glucose, 7.5 g/L (NH_4_)_2_SO_4_, 14.4 g/L KH_2_PO_4_, 0.5 g/L MgSO_4_·7H_2_O, 2 mL/L trace element solution and 50 μL/L antifoam (Sigma-Aldrich, St. Louis, MO, USA). After sterilization, vitamin solution was added at a concentration of 1 mL/L. The batch phase medium during aerated bioreactor cultivations contained 10 g/L glucose, 5 g/L (NH_4_)_2_SO_4_, 3 g/L KH_2_PO_4_, 0.5 g/L MgSO_4_·7H_2_O, 1 mL/L trace element solution, 50 μL/L antifoam and 1 mL/L vitamin solution. During the fed-batch phase, the feed medium was five times concentrated and contained glucose to a concentration of 100 g/L. The composition of the trace element and vitamin solution has been reported by Verduyn et al. [33].

### Shake flask cultivations

For cultivations in shake flasks, 5 mL of minimal medium were inoculated with a single colony from an agar plate with selective medium and incubated at 200 rpm and 30 °C for 48 h. Subsequently, the pre-culture was used to inoculate 18 mL of minimal medium in shake flasks without baffles and a total volume of 100 mL at an OD600 of 0.1. Finally, 2 mL of dodecane (≥99%, Reagent Plus, Sigma-Aldrich) were added to a final concentration of 10% v/v to sequester farnesene during the cultivation. Shake flasks were incubated at 180 rpm and 30 °C for 72 h.

### Bioreactor cultivations

For cultivation of *S. cerevisiae* strains in bioreactors, a single colony from a plate with selective medium was used to inoculate 5 mL of minimal medium used for shake flask cultivations. After incubation at 200 rpm and 30 °C overnight, the culture was transferred to a shake flask with 45 mL of minimal medium and incubated at 180 rpm and 30 °C for another 24 h. Subsequently, the culture was used to inoculate 400 mL of batch phase medium. Cultivations in bioreactors were performed using DasGip Parallel Bioreactor Systems for Microbiology (Eppendorf, Hamburg, Germany). The vessels were aerated at 30 L/h and homogeneous mixing was accomplished using a six-blade Rushton turbine at a stirring speed of 600 rpm. The temperature was adjusted to 30 °C and the pH was controlled at 5 using 2 M HCl and 2 M KOH. Composition of the exhaust gas was monitored on-line using the DasGip GA4 gas analyzer (Eppendorf). Bioreactor cultivations were started in batch mode. After glucose and ethanol depletion, 80 mL of dodecane were added under aseptic conditions and the feed was initiated. To allow for exponential growth at *μ* = 0.08 h^−1^, the feed rate was set to *ν*(*t*) = 0.003·exp(0.08·*t*), with *ν* as the feed rate in L/h and *t* as feed time in h. In addition, the feed was stopped at values of the respiratory quotient (*RQ*) above one to avoid overflow metabolism [32].

### BioLector cultivations

Growth curves were recorded using the BioLector (m2p-labs, Baesweiler, Germany). For this purpose, a single colony was picked from an agar plate with selective medium and used to inoculate 3 mL of minimal medium. After 48 h of incubation at 30 °C and 200 rpm, the culture was used to inoculate 1 mL of minimal medium at an OD600 of 0.1. Subsequently, the culture was transferred into a 48-well microtiter plate (MTP-48-B FlowerPlate, m2p-labs) and incubated at 30 °C and 1200 rpm using the BioLector. The optical density was measured on-line in 30 min intervals at a filter gain of 30.

### Measurement of cell growth

A spectrophotometer (Genesis20, Thermo Fisher Scientific, Waltham, MA, USA) was used to measure cell growth at the end of the shake flask cultivations. For cultivations in bioreactors, the biomass was measured by pipetting a 5 mL sample onto a pre-weighted filter with a pore size of 0.45 μm (Sartorius, Göttingen, Germany). The filter was rinsed with water, dried at 150 W for 15 min and kept in a desiccator until it was weighted again. Biomass concentrations are expressed as gram dry cell weight per volume (gDCW/L).

### Quantification of extracellular metabolites

For the quantification of glucose and ethanol, samples were taken at the end of the shake flask and during the bioreactor cultivations. For this purpose, the biomass was removed by centrifugation at 4000 rpm for 3 min or by filtration using a 0.45 μm nylon filter (VWR International AB, Stockholm, Sweden). Sample analysis was performed by HPLC using a Dionex Ultimate 3000 (Dionex, Sunnyvale, CA, USA) together with an Aminex HPX-87H column (300 × 7.8 mm, Bio-Rad Laboratories, Hercules, CA, USA) and a refractive index detector (512 μRIU). The column temperature was kept constant at 45 °C and 15 μL were injected into the mobile phase consisting of 5 mM H_2_SO_4_. The flow rate was set to 0.6 mL/min.

### Quantification of farnesene

At the end of the cultivation, the dodecane overlay was harvested by centrifugation at 4000 rpm for 3 min. Similarly, samples were collected during cultivations in bioreactors. Analysis was performed using a Focus GC-FID (Thermo Fisher Scientific) equipped with a ZB-50 column (Phenomenex, Torrance, CA, USA) as described previously with minor modifications [31]. The initial temperature was held at 50 °C for 1.5 min and then increased to 170 °C at a rate of 15 °C/min. After keeping the temperature constant for another 1.5 min, it was raised at the same rate to 300 °C and held for 3.0 min. The inlet temperature was set to 250 °C and 2 μL sample were injected in splitless mode. The base temperature of the flame was set to 300 °C. Farnesene was quantified using external calibration with *trans*-β-farnesene as analytical standard (≥90%, Sigma Aldrich). Samples were diluted in hexane and patchoulol (≥99%, a kind gift from Firmenich, Geneva, Switzerland) was added as internal standard. Concentrations of farnesene are stated based on the volume of the aqueous phase of the medium (mg/L_aq_).

### Quantification of lipids

Samples for lipid analysis were taken at the end of the fed-batch cultivations, after 15 h of feeding. For this purpose, 5 mL of the cultivation broth were added to 20 mL of methanol (Sigma-Aldrich), which was kept at −40 °C using a Huber CC-410 thermostat (Huber, Offenburg, Germany). Subsequently, the samples were centrifuged at 4000 rpm and −20 °C for 5 min. The supernatant was discarded and the cells were washed using 5 mL of methanol. After centrifugation at 4000 rpm and −20 °C for 5 min, the biomass was freeze-dried using a Christ alpha 2–4 LSC (Christ Gefriertrocknungsanlagen, Osterode, Germany) and analyzed as described by Khoomrung et al. [13].

## Results

### Heterologous expression of different *nphT7* homologs for farnesene production

**Fig. 2:**
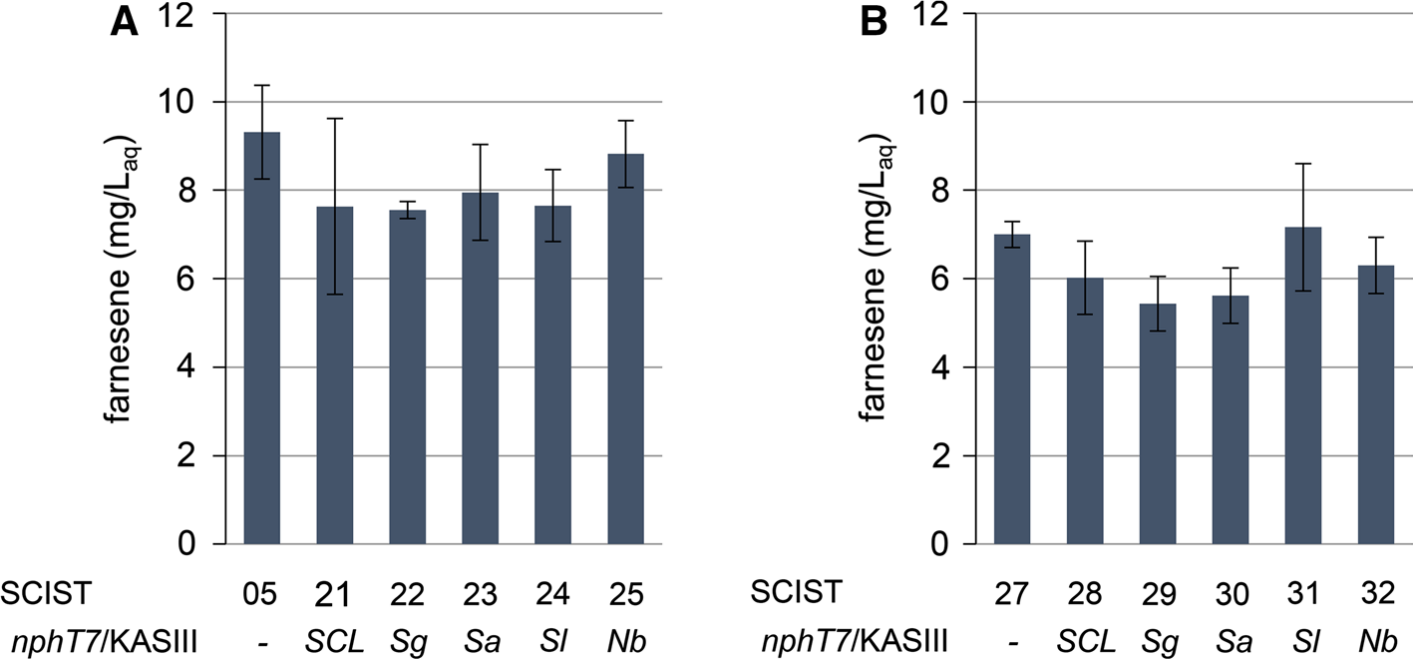
Effect of heterologous acetoacetyl-CoA synthase on production of farnesene in *S. cerevisiae*. Strains carry plasmids for expression of farnesene synthase, truncated HMG-CoA reductase and acetoacetyl-CoA synthase (*nphT7*) from different bacterial strains, without (**a**) and with (**b**) chromosomal integration of double mutant acetyl-CoA carboxylase (ACC1**). *SCL*—*Streptomyces* sp. strain CL190, *Sg*—*S. glaucescens*, *Sa*—*S. afghaniensis*, *Sl*—*S. lactacystinaeus* and *Nb*—*N. brasiliensis*. *Bars* represent average concentrations of farnesene with respect to the volume of aqueous medium of three biological replicates with standard deviation

Acetoacetyl-CoA synthase from *Streptomyces* sp. strain CL190 was identified to catalyze the irreversible decarboxylative condensation of malonyl-CoA and acetyl-CoA to acetoacetyl-CoA and could hence improve the production of isoprenoids. To determine the effect of the enzyme on the production of sesquiterpenes, *nphT7*_*SCL*_ was expressed from a multi-copy plasmid together with a farnesene synthase from *Citrus junos* (*FarnSyn*), which converts FPP to farnesene [16] and a truncated version of the HMG-CoA reductase (*tHMG1*), which reduces HMG-CoA to mevalonate, but lacks the NH_2_-terminal transmembrane domain [8, 24]. The structure of plasmid pIST12 is presented in Fig. S7 of the supplementary material. In addition, four homologous genes from other bacterial species, i.e. *S. glaucescens*, *S. afghaniensis, S. lactacystinaeus* and *N. brasiliensis*, were selected to identify differences regarding the efficiency of the enzymes. The first two genes encode for putative acetoacetyl-CoA synthases, while the latter two belong to the class of β-ketoacyl-ACP synthase (KAS) III enzymes, which convert malonyl-ACP and acetyl-CoA to acetoacetyl-ACP. Although *nphT7*_*SCL*_ is homologous to *fabH* (KASIII) from *E. coli*, also containing the highly conserved catalytic triad at Cys115, His256 and Asn286, the enzyme did not show KASIII activity, presumably because it lacks an important arginine residue required for ACP binding [21]. On the contrary, KASIII enzymes from *S. lactacystinaeus* and *N. brasiliensis* share an AGGSR motif with the amino acid sequence of NphT7, which is not present in FabH from *E. coli*, but was suggested to be involved in recognition of the CoA moiety [21]. Hence, these enzymes could potentially be able to accept malonyl-CoA as substrate. Multiple sequence alignment of the homologs showing the putative CoA binding motif and results of a BLAST of these homologs to NphT7 from *Streptomyces* sp. strain CL190 are presented in Fig. S1 and Table S3 of the supplementary material. When *S. cerevisiae* strain CEN.PK113-5D was transformed with plasmids pIST12-16 and cultivated in shake flasks, expression of *nphT7*_*SCL*_ from *Streptomyces* sp. strain CL190 did not improve production of farnesene in comparison to strain SCIST05, which does not express an acetoacetyl-CoA synthase (Fig. 2a). In fact, final farnesene titers slightly decreased on average from 9.3 to 7.6 mg/L_aq_. Furthermore, no difference between the strains expressing the different homologs was observed as farnesene concentrations were almost equal with approximately 8 mg/L_aq_. Since the effect of the enzymes could be restricted by the supply of malonyl-CoA, which is a crucial intermediate in fatty acid synthesis, we aimed at increasing the availability of malonyl-CoA as extender substrate for the acetoacetyl-CoA synthase. For this reason, plasmids pIST12-16 were also introduced into strain SCIST26, which carries a chromosomal integration of the mutated acetyl-CoA carboxylase (ACC1**) (Fig. 2b). Site mutations at Ser659 and Ser1157 have been shown to reduce sensitivity to Snf1 mediated phosphorylation, which led to a substantial increase in Acc1p activity [30]. However, even with the expression of ACC1** there was no improvement of farnesene production and no difference between the different homologs. Surprisingly, final farnesene titers were reduced overall to approximately 6 mg/L_aq_.

### Evaluation of an alternative pathway for farnesene production

**Fig. 3:**
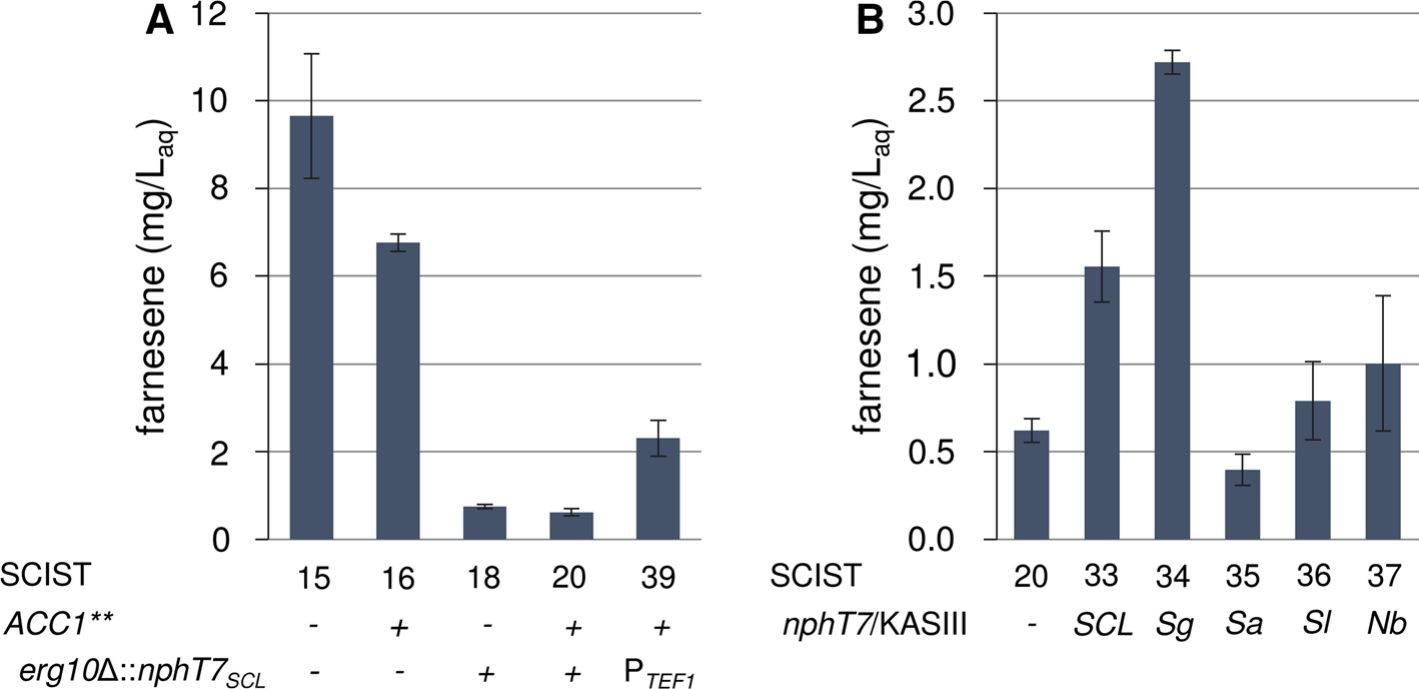
**a** Replacing endogenous *ERG10* in *S. cerevisiae* by *nphT7* from *Streptomyces* sp. strain CL190 in combination with ACC1** overexpression for production of farnesene. All strains carry plasmid pIST05 for expression of farnesene synthase and truncated HMG-CoA reductase. **b** Comparison of different *nphT7* homologs in strain SCIST19 (ACC1**, *erg10*Δ::*nphT7*_*SCL*_). The homologs were expressed from plasmid together with farnesene synthase and truncated HMG-CoA reductase. *SCL*—*Streptomyces* sp. strain CL190, *Sg*—*S. glaucescens*, *Sa*—*S. afghaniensis*, *Sl*—*S. lactacystinaeus* and *Nb*—*N. brasiliensis*. *Bars* represent average concentrations of farnesene with respect to the volume of aqueous medium of at least three biological replicates with standard deviation

Overexpression of *nphT7*_*SCL*_ did not result in improved production of farnesene. Promoted thiolysis of acetoacetyl-CoA to acetyl-CoA due to increased levels of acetoacetyl-CoA could explain these results. To confirm this observation, we aimed at providing acetoacetyl-CoA exclusively via the malonyl-CoA route, allowing for a comparison of the native and the modified pathway. Therefore, endogenous *ERG10* was replaced in frame by *nphT7*_*SCL*_, leaving the *ERG10* promoter and terminator. While the endogenous pathway is limited by thermodynamic constraints, the bypass via malonyl-CoA requires an additional reaction and increases the ATP demand of the pathway. Although ATP conservation plays a crucial role in metabolic engineering and an increased ATP demand is preferably avoided [6], ATP has recently been used as a driving force for the production of butanol in *S. elongatus* PCC 7942 [14]. Substitution of *ERG10* by *nphT7*_*SCL*_ was performed in strains with and without ACC1** integration. Although the increased conversion of acetyl-CoA to malonyl-CoA using ACC1** by itself had a negative effect on production of farnesene (Fig. 2b), more flux towards malonyl-CoA might be beneficial to support the pathway when *ERG10* is removed. However, in both strains (SCIST18 and 20), the loss of *ERG10* resulted in a substantial decrease of final farnesene titers by more than 90%, whereas titers were slightly higher without overexpression of ACC1** (Fig. 3a). Since both strains utilize the endogenous *ERG10* promoter and terminator, these results indicate that the expression of *nphT7*_*SCL*_ is not sufficient to fully compensate for the loss of *ERG10*. Variation of the promoter strength is commonly used to improve expression [1]. Likewise, the terminator sequence has been shown to alter expression by changing mRNA stability [4]. Hence, integration of the gene was also performed using P_*TEF1*_ and T_*ADH1*_ to enhance the transcript level (strain SCIST39). Although farnesene concentrations remained significantly lower compared to the reference (SCIST05), manipulation of the *nphT7*_*SCL*_ expression level resulted in an almost fourfold increase of farnesene production compared to SCIST20 (ACC1**, *erg10*Δ::*nphT7*_*SCL*_ with pIST05) (Fig. 3a). To evaluate the selected homologs when acetoacetyl-CoA cannot be degraded by acetoacetyl-CoA thiolase, plasmids pIST12-16 carrying the different *nphT7*_*SCL*_ homologous genes, were introduced into strain SCIST19 (ACC1**, *erg10*Δ::*nphT7*_*SCL*_). Similar to varying the promoter strength of the chromosomally integrated *nphT7* from *Streptomyces* sp. strain CL190, a higher copy number (and using a strong promoter) also led to higher final farnesene titers (SCIST33, Fig. 3b). Furthermore, a clear variation between the different homologs was observed, where expression of acetoacetyl-CoA synthase from *S. glaucescens* improved production fourfold compared to SCIST20. Surprisingly, a decrease in production was observed for acetoacetyl-CoA synthase from *S. afghaniensis*. Overexpression of KASIII enzymes from *S. lactacystinaeus* and *N. brasiliensis* did not have a significant effect on production of farnesene.

**Fig. 4:**
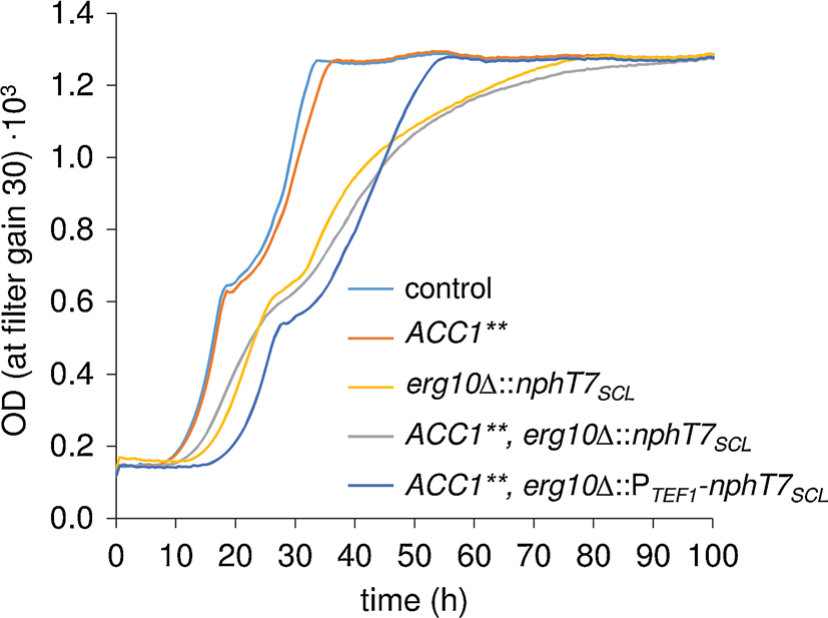
Effect of replacing endogenous *ERG10* by bacterial *nphT7*_*SCL*_ on growth of *S. cerevisiae.* Average optical density of four biological replicates measured online using BioLector. Indicated relevant genotypes refer to strains: SCIST15 (control), 16, 18, 20, 39 (from *top* to *bottom*)

Considering the final optical density, the growth of the strains expressing *nphtT7*_*SCL*_ instead of *ERG10* was clearly affected (Fig. S2). While SCIST15 (control, CEN.PK113-5D background) and SCIST16 (ACC1**) reached an equal final OD600 of 12.4 after 72 h of cultivation, strains SCIST18 (*erg10*Δ::*nphT7*_*SCL*_) and SCIST20 (ACC1**, *erg10*Δ::*nphT7*_*SCL*_) reached a final OD600 of 5.5 and 7.2 on average, respectively. In accordance with this, the cultivation broth of strain SCIST18 and 20 still contained 5.0 and 3.6 g/L of ethanol. On the other hand, final OD and ethanol concentration were almost identical when expression of *nphT7*_*SCL*_ was enhanced using P_*TEF1*_ (SCIST39). This observation prompted us to investigate the growth kinetics in more detail. For this purpose, strains SCIST15, 16, 18, 20 and 39 were cultivated using the BioLector to monitor growth continuously. From the recorded growth curves shown in Fig. 4 it can be seen that strains SCIST15 and 16 entered the diauxic shift after approximately 20 h of cultivation and reached stationary phase after approximately 35 h. In contrast, for strains SCIST18 and 20 the diauxic shift occurred after approximately 30 h and consumption of all carbon sources from the medium required up to 90 h. Especially during the second half of the cultivation, while the control (SCIST15) was still growing exponentially, these strains displayed significantly impaired growth, which could point towards transcriptional regulation of *ERG10* during the ethanol consumption phase. Strain SCIST39 (ACC1**, *erg10*Δ::P_*TEF1*_-*nphT7*_*SCL*_) with enhanced expression of *nphT7*_*SCL*_ using P_*TEF1*_ displayed a longer lag phase, but otherwise similar growth in comparison to the reference. To quantify the effect of the *ERG10* substitution by *nphT7*_*SCL*_ the specific growth rates were calculated for exponential growth during each phase (Fig. S3), which demonstrates a clear reduction that can be assigned to this modification. Finally, the identical experiment was also performed with strains SCIST33-37 (ACC1**, *erg10*Δ::*nphT7*_*SCL*_), which express the different homologs in addition to the integrated *nphT7* from *Streptomyces* sp. strain CL190 (Fig. S4) to examine their effect on growth. In accordance with the results for final farnesene titers (Fig. 3b), *nphT7* from *S. glaucescens* showed the most significant effect on growth, particularly during the ethanol consumption phase in comparison to the reference SCIST20 (ACC1**, *erg10*Δ::*nphT7*_*SCL*_) as stationary phase was reached after 60 h instead of over 100 h of cultivation.

### Manipulating the pool of malonyl-CoA by downregulation of fatty acid synthesis

**Fig. 5:**
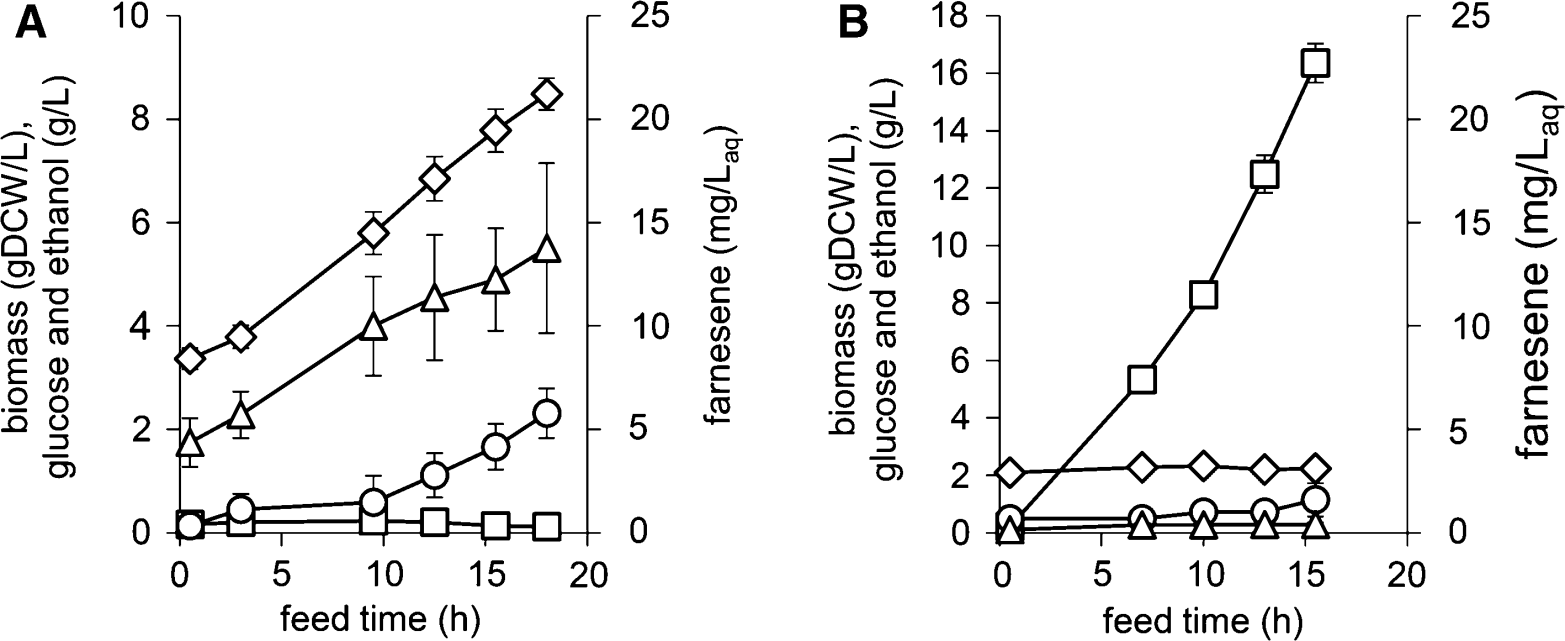
Downregulation of *FAS1* using P_*HXT1*_ in *S. cerevisiae* with ACC1** overexpression and *erg10*Δ::P_*TEF1*_-*nphT7*_*SCL*_ substitution from *Streptomyces* sp. strain CL190 for improved utilization of malonyl-CoA as substrate for the mevalonate pathway. **a** Strain SCIST40 without *FAS1* downregulation and **b** strain SCIST43 with *FAS1* downregulation. In addition to the integrated copy, both strains express *nphT7* from *S. glaucescens* from plasmid pIST13. Data represents average values of four biological replicates with standard deviation obtained during the fed-batch phase. *Diamonds* biomass, *squares* glucose, *circles* ethanol and *triangles* farnesene

Farnesene concentrations decreased substantially after replacing acetoacetyl-CoA thiolase by the respective acetoacetyl-CoA synthase from *Streptomyces* sp. strain CL190. Malonyl-CoA represents an important precursor for this pathway as well as for fatty acid synthesis and, therefore, its availability for the mevalonate pathway might be insufficient. Since overexpression of ACC1** alone did not improve pathway efficiency, downregulation of fatty acid synthase could serve as a potential strategy to redirect more flux towards the mevalonate pathway. For this purpose, P_*HXT1*_ was used to downregulate *FAS1*, encoding the beta subunit of the fatty acid synthase in yeast, which utilizes acetyl-CoA and malonyl-CoA to synthesize fatty acyl-CoA. The hexose transporter Hxt1p was shown to be active in *S. cerevisiae* at higher glucose concentrations (≥1% [26], full induction at 4% [22]) and its promoter has previously been used to increase the production of sesquiterpenes by diverting flux from the FPP branch point [28]. For this reason, strains were cultivated in fed-batch mode to obtain low glucose concentrations and to activate downregulation of *FAS1*. The feeding profile was designed to allow for exponential growth and to keep the glucose concentration close to zero. With the objective to reduce overflow metabolism and to maintain fully respiratory conditions, the respiratory quotient (*RQ*) was additionally used as a control parameter to indirectly control the concentration of glucose in the medium [32]. To utilize the available pool of malonyl-CoA more efficiently as a substrate for the mevalonate pathway, strain SCIST41 (ACC1**, *erg10*Δ::P_*TEF1*_-*nphT7*_*SCL*_, P_*FAS1*_Δ::P_*HXT1*_) was transformed with plasmid pIST13 carrying *nphT7* from *S. glaucescens*, which showed higher efficiency compared to the one from *Streptomyces* sp. strain CL190 (Fig. 3b). Strain SCIST40 (ACC1**, *erg10*Δ::P_*TEF1*_-*nphT7*_*SCL*_) did not contain the modification of *FAS1* and was used as a reference. The two strains showed significantly different growth profiles during the batch phase as indicated by their CO_2_ profiles, showing that downregulation of *FAS1* resulted in reduced CO_2_ formation during the glucose and ethanol phase (Fig. S5). During the fed-batch phase the medium was fed exponentially to theoretically support a specific growth rate of *μ* = 0.08 h^−1^. However, both strains did not maintain growth at 0.08 h^−1^, with strain SCIST40 reaching a growth rate of 0.068 ± 0.003 h^−1^. Ethanol reached 2.3 g/L on average and the glucose concentration remained close to zero over 18 h (Fig. 5a). After approximately 12 h, the *RQ* control was activated, which was used as feedback control to avoid overflow metabolism, resulting in slightly oscillating *RQ* values (Fig. S6). Farnesene was produced at a yield of 0.57 ± 0.19 mg/g glucose with final titers of 13.8 ± 4.1 mg/L_aq_. On the contrary, the percentage of CO_2_ in the exhaust gas slightly decreased over time for strain SCIST43 (Fig. S6) and biomass concentrations stayed almost constant at approximately 2.2 gDCW/L (Fig. 5b). Farnesene and ethanol could only be detected in minor quantities. Consistent with these data, the glucose concentration increased, reaching up to 16.4 ± 0.7 g/L. To identify changes in fatty acid metabolism due to downregulation of *FAS1*, the lipid content of the cell was analyzed at the end of the fed-batch. The most significant differences were observed considering the content of storage lipids as triacylglycerides dropped by 88% from 31.6 ± 8.5 to 3.9 ± 0.4 mg/gDCW (Table 3). Besides, a clear effect was observed regarding the membrane composition as phosphatidylcholine, phosphatidylinositol as well as ergosterol decreased substantially.

**Table 3:** Lipid quantification in strains SCIST40 and 43 after 15 h of exponential feeding

	−*FAS1* downregulation (SCIST40)	+*FAS1* downregulation (SCIST43)
Triacylglycerol	31.56 ± 8.49	3.89 ± 0.41
Phosphatidylinositol	2.47 ± 0.50	0.46 ± 0.07
Phosphatidylcholine	2.33 ± 0.45	0.80 ± 0.10
Ergosterol	3.43 ± 0.60	1.72 ± 0.21

Data represent average values in mg/gDCW of four biological replicates ± standard deviation

## Discussion

A recently identified acetoacetyl-CoA synthase from *Streptomyces* sp. strain CL190, which catalyzes the condensation of malonyl-CoA and acetyl-CoA to generate acetoacetyl-CoA, has been proposed as a potential target to enhance production of acetoacetyl-CoA derived compounds. In this study, the encoding gene, *nphT7*, was expressed in *S. cerevisiae* and its effect on cell growth and production of farnesene was investigated. Besides, different homologs were compared with the objective to identify an acetoacetyl-CoA synthase with superior efficiency. For this purpose, *nphT7*_*SCL*_ and the respective homologs from *S. glaucescens*, *S. afghaniensis*, *S. lactacystinaeus* and *N. brasiliensis* were expressed from plasmid together with genes encoding a farnesene synthase (*FarnSyn*) and a truncated version of the HMG-CoA reductase (*tHMG1*), which has been identified as a flux controlling enzyme of the mevalonate pathway [20]. Most importantly, production of farnesene did not benefit from acetoacetyl-CoA synthase expression in these experiments (Fig. 2a). In addition, no difference was observed between yeast strains expressing the selected homologs from other bacterial species considering the final titers of farnesene. In fact, final titers appeared to be slightly decreased in comparison to the reference (no expression of *nphT7*_*SCL*_, SCIST05). While these results could indicate insufficient activity of NphT7, the levels of malonyl-CoA were assumed to play a crucial role for this route and the overall effect of the enzyme. To investigate this hypothesis, the intracellular concentration of malonyl-CoA was increased by overexpression of ACC1** [30]. However, raising the pool of malonyl-CoA revealed to have a negative effect on the production of farnesene (Fig. 2b). Apart from this, expression of *tHMG1* was used to pull the flux towards the downstream reactions of the pathway and to assure efficient conversion of acetoacetyl-CoA. Nonetheless, higher concentrations of acetoacetyl-CoA may promote the thiolysis activity of the endogenous Erg10, ultimately generating a futile cycle between acetyl-, malonyl- and acetoacetyl-CoA. Therefore, we aimed at blocking thiolysis activity of acetoacetyl-CoA thiolase by replacing *ERG10* with *nphT7* from *Streptomyces* sp. strain CL190. This step is critical as deletion of *ERG10* was shown to result in mevalonate auxotrophy [10]. Although *nphT7*_*SCL*_ was able to complement the loss of *ERG10*, significant changes were observed considering growth and product formation. Strains with *nphT7*_*SCL*_ integrations displayed slower growth, particularly during the ethanol consumption phase (Fig. 4). Similarly, production of farnesene was strongly impaired as final titers decreased by 92% (SCIST15 vs. SCIST18, Fig. 3a), illustrating that the availability of acetoacetyl-CoA was severely impaired when the endogenous thiolase was lost. To enhance the flux via the malonyl-CoA bypass, this strategy was again combined with overexpression of ACC1**. However, consistent with previous results, increasing the level of malonyl-CoA did not improve farnesene production. Altogether, these results illustrate that the bypass via malonyl-CoA is significantly less efficient compared to the endogenous pathway. Clearly, production of FPP is not only extended by an additional reaction in the altered pathway, the demand of ATP also increases from 9 to 12 mol of ATP per mole of FPP due to the conversion of acetyl-CoA to malonyl-CoA, which makes the pathway disadvantageous from an energetic perspective. However, sufficient flux towards malonyl-CoA is essential to maintain growth as it represents an essential building block in lipid metabolism. Instead of the greater ATP cost of the pathway, insufficient efficiency of the acetoacetyl-CoA synthase could cause these detrimental effects. To further investigate this hypothesis, two approaches were pursued to enhance expression of *nphT7*_*SCL*_. First, a different promoter (P_*TEF1*_) was used for expression instead of the *ERG10* promoter, which resulted in an almost fourfold increase (Fig. 3a, SCIST20 vs. SCIST39). Although data on the activity of the promoters in these conditions is not available, transcriptional regulation of *ERG10* could be impaired when P_*TEF1*_ is used for expression [7]. Second, the copy number was increased by expressing the gene from a multi-copy plasmid in addition to the integrated copy (also using a strong promoter). In this case, a more than twofold increase in final farnesene titers was detected (Fig. 3b, SCIST20 vs. SCIST33). Surprisingly, a difference between the selected homologs became apparent, clearly indicating superior efficiency of *nphT7* from *S. glaucescens*, leading to a fourfold increase (Fig. 3b, SCIST20 vs. SCIST34). Similarly, Lan and Liao [14] investigated the in vitro activity of different NphT7 homologs, e.g. from *Streptomyces coelicolor*, *Streptomyces avermitilis* and *Pseudomonas aeruginosa*, but found NphT7 from *Streptomyces* sp. strain CL190 to be the most active. Besides farnesene production, also the growth kinetics could be improved by enhancing the expression or increasing the copy number. Except for the extended lag phase, SCIST39 (P_*TEF1*_-ACC1**, *erg10*Δ::P_*TEF1*_-*nphT7*_*SCL*_) almost showed the same growth profile as SCIST15 (control) (Fig. 4). Similarly, SCIST34, which expresses *nphT7* from *S. glaucescens* from plasmid showed the most significant improvement in growth compared with SCIST20 (Fig. S4). Based on these results, we conclude that acetoacetyl-CoA synthase activity is not sufficient to enable high fluxes through the mevalonate pathway. This could also partially explain why no improvement was attained when levels of malonyl-CoA were elevated by overexpression of ACC1**. On the other hand, malonyl-CoA is the key precursor for lipid synthesis and enhancing its concentration may divert the flux away from the mevalonate pathway. Therefore, downregulation of *FAS1* aimed at increasing the availability of this substrate, which might otherwise be lost to competing reactions. This approach was recently used to significantly improve production of 3-HP, which is directly derived from malonyl-CoA [5]. Previous studies have shown that the activity of the *HXT1* promoter amounts to less than half of the *FAS1* promoter at glucose concentrations of 2% [12], which was clearly visible during the batch phase (Fig. S5) and as anticipated, even more pronounced during the fed-batch phase (Fig. 5b, Fig. S6). Efficient downregulation of *FAS1* was also evident by the significant reduction of storage lipids (TAGs) and phospholipids (Table 3), which suggests increased availability of malonyl-CoA in connection with ACC1** overexpression. However, instead of using the pool of malonyl-CoA for the mevalonate pathway to a greater extend, the metabolic activity of the strain was significantly reduced as seen by the CO_2_ profile as well as the production of farnesene and ethanol (Fig. 5b, Fig. S6). While these results again point towards the low efficiency of NphT7, this phenotype could potentially arise from a build-up of malonyl-CoA to toxic levels, a mechanism, which has previously been studied in human cancer cells [23]. Here, inhibition of the fatty acid synthase was shown to be severely toxic, which was accompanied by a substantial increase in the intracellular levels of malonyl-CoA. Surprisingly, although starvation for fatty acids served as a potential explanation for these results, inhibition of the preceding reaction catalyzed by acetyl-CoA carboxylase did not impair the viability of the cells and resulted in a reduction of malonyl-CoA concentrations.

In conclusion, this study investigated utilization of *nphT7* from *Streptomyces* sp. strain CL190 as a target to improve production of farnesene. Similar studies have been performed for example for the production of butanol with diverse outcomes. In *E. coli*, overexpression of the *nphT7* route was significantly less efficient for acetoacetyl-CoA supply compared with the *atoB* route [19]. Schadeweg et al. on the other hand could show that NphT7 supports production of butanol [29]. In our experiments, expression of *nphT7*_*SCL*_ did not improve production. We could show that the efficiency of the enzyme represents a central aspect that affects the functionality of the overall pathway. Although *nphT7*_*SCL*_ was able to compensate for the loss of *ERG10*, which otherwise would result in an auxotrophy for mevalonate, the replacement had a severe effect on cell growth. Laboratory evolution could be used in the future as a strategy to improve growth of the strain. Furthermore, expressing different homologs of *nphT7*_*SCL*_ in the *erg10*Δ background allowed for identification of a superior candidate from *S. glaucescens*. To overcome disadvantageous properties of acetoacetyl-CoA synthase, several alternative strategies exist to improve flux through the first part of the pathway. Production of sesquiterpenes could be significantly enhanced by improving acetyl-CoA synthesis in combination with overexpression of the endogenous *ERG10* [2]. On the other hand, acetoacetyl-CoA thiolase from *Clostridium acetobutylicum*, which has been previously used for production of farnesene [27], was shown to have a significantly higher in vitro activity compared to the thiolase from *S. cerevisiae* [29]. Besides, further genetic modifications might be required to pull more flux through the mevalonate pathway and particularly to redirect flux from the FPP branch point towards farnesene.

## Electronic supplementary material

Below is the link to the electronic supplementary material.

Supplementary material 1 (DOCX 1995 kb)
