# Supplementary material for: Effects of acetoacetyl-CoA synthase expression on production of farnesene in Saccharomyces cerevisiae
Source: J Ind Microbiol Biotechnol. 2017 Feb 9;44(6):911–22. doi: 10.1007/s10295-017-1911-6 (PMC5432608; doi:10.1007/s10295-017-1911-6)
Supplement: Supplementary file 1 — Supplementary material 1 (DOCX 1995 kb) [file 10295_2017_1911_MOESM1_ESM.docx]

# Supplementary Material

# Effects of acetoacetyl-CoA synthase expression on production of farnesene in *Saccharomyces cerevisiae*

Stefan Tippmann^1,2^, Raphael Ferreira^1,2^, Verena Siewers^1,2^, Jens Nielsen^1,2,3^ and Yun Chen^1,2,^*

^1^Department of Biology and Biological Engineering,

Chalmers University of Technology, SE412 96 Gothenburg, Sweden

^2^Novo Nordisk Foundation Center for Biosustainability, Chalmers University of Technology, SE412 96 Gothenburg, Sweden

^3^Novo Nordisk Foundation Center for Biosustainability, Technical University of Denmark, DK2800 Kgs. Lyngby, Denmark

*Corresponding Author: Yun Chen

Department of Biology and Biological Engineering,
Chalmers University of Technology, Kemivägen 10, SE412 96, Gothenburg, Sweden.

E-mail: yunc@chalmers.se

Tel: +46 31772 38 04

Fax: +46 31 772 38 01

Keywords: isoprenoids, mevalonate pathway, biofuels, yeast, metabolic engineering

Table S1 List of primers used for plasmid construction. Italics indicate overhangs, bold letters indicate restriction sites.

| Plasmid | Primer Name | Sequence (5’ 🡪 3’) |
| --- | --- | --- |
| pIST07 | SCL190-fwd | *GTTGTT****GCGGCCGC***AAAACAATGACCGATGTTAGATT |
|  | SCL190-rev | *GTTGTT****TTAATTAA***TTACCATTCAATCAAGGCAA |
| pIST12/13/ 14/15/16 | PHXT7-fwd | *GGAATTGCCATGAAGCCGAA*TCGTAGGAACAATTTCGGG |
|  | PHXT7-rev | TTTTTGATTAAAATTAAAAAAACTTTTTG |
|  | THIS5-fwd | ATAGATTAATTTAAACAGTATATGTACAG |
|  | THIS5-rev | GTAACAATATCATGAGACCTTTTATA |
| pIST12 | nphT7-fwd | *TTTTTAATTTTAATCAAAAA*AAAACAATGACCGATGTTAGATTCAGA |
|  | nphT7-rev | *TACTGTTTAAATTAATCTAT*TTACCATTCAATCAAGGCA |
| pIST13 | Sgl-fwd | *TTTTTAATTTTAATCAAAAA*AAAACAATGATTACTACAGGTACTC |
|  | Sgl-rev | *TACTGTTTAAATTAATCTAT*TTATCTTGTTAACAATGCCA |
| pIST14 | Saf-fwd | *TTTTTAATTTTAATCAAAAA*AAAACAATGGCTGCATCTAC |
|  | Saf-rev | *TACTGTTTAAATTAATCTAT*TTATCTATTACCCCAACTAATTAA |
| pIST15 | Sla-fwd | *TTTTTAATTTTAATCAAAAA*AAAACAATGACCGATGTTAG |
|  | Sla-rev | *TACTGTTTAAATTAATCTAT*TTACCATTCAACTAAAGCGA |
| pIST16 | Nbr-fwd | *TTTTTAATTTTAATCAAAAA*AAAACAATGAATAACATTGCTG |
|  | Nbr-rev | *TACTGTTTAAATTAATCTAT*TTACCATTCGACTAAAGTCAAA |
| pIST12/13/ 14/15/16 | T7Cassette-fwd | *GGCGTTTATCCAGCTGCATTAATGA*TCGTAGGAACAATTTCGGG |
|  | T7Cassette-rev | *CTGATTCTGTGGATAACCGTATTACC*GTAACAATATCATGAGACCTTTTATA |
|  | Affi3-2-fwd | GGTAATACGGTTATCCACAGAATC |
|  | Affi3-2-rev | TTCTCAGGTATAGCATGAGGTC |
|  | Affi3-3-fwd | GAGCGACCTCATGCTATACCT |
|  | Affi3-3-rev | TCATTAATGCAGCTGGATAAAC |

Table S2 Primers for replacing *Erg10* by *nphT7_SCL_* using CRISPR/Cas9.

| Repair fragment | Primer Name | Sequence (5' 🡪 3') |
| --- | --- | --- |
| *nphT7_SCL_* | NphRF-fwd | *AAAGGTAGCCTAAAACAAGCGCCATATCATATATATTTATACAGATTAGACGTACTCAAA*  AAAACAATGACCGATGTTAGATTC |
|  | NphRF-rev | *AAGCCATTTATATATTTATGTATTTTATGAAAAAGATCATGAGAAAATCGCAGAACGTAA*  TTACCATTCAATCAAGGCA |
| P*_TEF1_*-*nphT7_SCL_*-T*_ADH1_* | NphRF2-fwd | *AAAGGTAGCCTAAAACAAGCGCCATATCATATATATTTATACAGATTAGACGTACTCAAA*  GCACACACCATAGCTTCAAA |
|  | NphRF2-rev | *AAGCCATTTATATATTTATGTATTTTATGAAAAAGATCATGAGAAAATCGCAGAACGTAA*  GAGCGACCTCATGCTATACC |

Table S3 BLAST results to compare the amino acid sequences of the NphT7 homologs to NphT7 from *Streptomyces* sp. strain CL190.

| Origin | Accession number | E-value | Identity (%) |
| --- | --- | --- | --- |
| *Streptomyces glaucescens* | AIR99429.1 | 2e-120 | 60 |
| *Streptomyces afghaniensis* | WP_020277513.1 | 4e-103 | 56 |
| *Streptomyces lactacystinaeus* | BAP82212.1 | 7e-175 | 75 |
| *Nocardia brasiliensis* | GAJ84426.1 | 4e-142 | 66 |

Fig. S1 Sequence alignment of amino acid sequences for *nphT7* and four different homologs using CLUSTALW 2.1 on (http://www.genome.jp/tools/clustalw/). Region highlighted in grey indicates the motif potentially involved in CoA recognition.

Fig. S2 Ethanol concentration and final OD600 values after 72 h of cultivation after replacing endogenous *ERG10* in *S. cerevisiae* by *nphT7* from *Streptomyces* sp. CL190 in combination with *ACC1*** overexpression. Bars represent average values of at least three biological replicates with standard deviation. The corresponding final titers of farnesene are shown Fig. 3A.

Fig. S3 Specific growth rates calculated during the glucose and ethanol phase using the OD measurements from the BioLector cultivation presented in Fig. 4.

Fig. S4 Effect of different *nphT7* from various bacterial species on growth of *S. cerevisiae.* Average optical density of three biological replicates measured online using BioLector. All genes were expressed from plasmid in strain SCIST19 (*ACC1***, *erg10*∆::*nphT7_SCL_*). Strain SCIST20 was used as control.

Fig. S5 CO_2_ profile for four biological replicates of strain SCIST40 (A) and SCIST43 (B) during aerobic batch cultivation using 10 g/L glucose. SCIST40 - P*_TEF1_*-*ACC1***, *erg10*∆::P*_TEF1_*- *nphT7_SCL_* and SCIST43 - P*_TEF1_*-*ACC1***, *erg10*∆::P*_TEF1_*- *nphT7_SCL_*, P*_FAS1_*∆::P*_HXT1_*.

Fig. S6 CO_2_ and respiratory quotient (*RQ*) profiles for four biological replicates of strain SCIST40 (A-1, A-2) and SCIST43 (B-1, B-2) during the fed-batch phase of aerobic cultivations in bioreactors using exponential feeding. SCIST40 - P*_TEF1_*-*ACC1***, *erg10*∆::P*_TEF1_*- *nphT7* and SCIST43 - P*_TEF1_*-*ACC1***, *erg10*∆::P*_TEF1_*- *nphT7*, P*_FAS1_*∆::P*_HXT1_*.

Fig. S7 Plasmid map of pIST12 for expression of farnesene synthase from *Citrus junos* (*FarnSyn_Cj*), truncated HMG-CoA reductase (*tHMG1*) and acetoacetyl-CoA synthase (*nphT7*) from *Streptomyces* sp. strain CL190. Plasmid pIST13-16 are identical, but express *nphT7* homologs from other bacterial strains.
